# Catecholamines and Neurodegeneration in Parkinson’s Disease—From Diagnostic Marker to Aggregations of α-Synuclein

**DOI:** 10.3390/diagnostics3020210

**Published:** 2013-03-25

**Authors:** Hideyuki Sawada, Tomoko Oeda, Kenji Yamamoto

**Affiliations:** Clinical Research Center, Utano National Hospital, National Hospital Organization, 8 Ondoyama-cho, Narutaki, Ukyoku, Kyoto 616-8255, Japan; E-Mails: mlb36641@nifty.com (T.O.); kyama1024@yahoo.co.jp (K.Y.)

**Keywords:** α-synuclein, aggregation, biomarker, neuroprotection, disease-modifying

## Abstract

Parkinson’s disease is the second most prevalent disease of the brain. It is characterized by midbrain dopaminergic neuronal degeneration accompanied by Lewy bodies, intra-cytoplasmic neuronal inclusions that consist mainly of alpha-synuclein. The cardinal motor features are muscular rigidity, bradykinesia, and resting tremor and, in advanced cases, postural instability. Symptoms are relieved by dopamine replacement therapy, but progress slowly. Clinical diagnosis is made according to medical history, neurological examinations and the response to anti-Parkinsonian drugs. There are no laboratory tests for diagnosis of the disease; however, for development of disease-modifying treatment, early diagnosis by objective laboratory test is required. Recently, postsynaptic sympathetic norepinephrine nerve terminals were found to be degenerated as well as mesencephalic dopaminergic neurons. Cardiac norepinephrine denervation can be seen by meta-iodine-benzyl guanidine scintigraphy, and may be a reliable diagnostic marker. Degeneration of norepinephrinergic and dopaminergic neurons suggests that catecholamines may play a central role in the neurodegeneration in Parkinson’s disease. Recently several studies showed that alpha-synuclein aggregates in cells exposed to dopamine. Here, we review findings relating to an early diagnostic marker for detecting degeneration of the peripheral sympathetic nerves, and propose the hypothesis that catecholamines cause alpha-synuclein to aggregate and play an important role in disease pathogenesis.

## 1. Introduction

Parkinson’s disease is characterized by mesencephalic nigral dopaminergic neuronal degeneration accompanied by Lewy bodies, intra-cytoplasmic neuronal inclusions that consist mainly of insoluble aggregated alpha-synuclein [1,2,3]. It usually occurs in the sixth decade of life, or later, and the prevalence has been increasing together with the aging of the population in European countries [4]. Cardinal motor features are bradykinesia, muscular rigidity and resting tremor that are caused by dopamine depletion in the striatum owing to a loss of projections from mesencephalic dopaminergic neurons. Laboratory examinations, including blood tests and brain MRI, reveal no disease-specific findings; therefore, a clinical diagnosis is made according to history, neurological examinations and the response to dopamine replacement therapy [5]. In addition to motor disturbances, non-motor symptoms and signs such as orthostatic hypotension, sleep disturbance, anhedonia, cognitive decline, and hallucinations are seen in Parkinson’s disease [6]. Cognitive decline or hallucinations occur usually in advanced stages of the disease, and in contrast, several non-motor symptoms including REM sleep behavioral disturbance [7], constipation or hyposmia precede motor symptoms [8].

When striatal dopamine is depleted by 60–70%, clinical motor symptoms appear. The preclinical stage, in which dopaminergic neuronal degeneration precedes the emergence of motor symptoms, seems to last for years [9]. Therefore, it is thought that dopaminergic neurons have already degenerated moderately when the diagnosis of Parkinson’s disease is made. Several recent clinical trials have investigated the drug effects in terms of modifying disease progression, although their results are controversial [10,11]. The presence of a laboratory diagnostic marker for early diagnosis or preclinical diagnosis of Parkinson’s disease would make it possible to design a better disease-modifying model. Here, potential laboratory biomarkers for the diagnosis of Parkinson’s disease, including olfactory disturbance [12,13], cardiac MIBG scintigram [14], and eye drop tests using cocaine and phenylephrine [15] are reviewed.

## 2. Possible Disease-Modifying Effects and Diagnostic Tests for Detecting Early-Stage Disease

**Figure 1:**
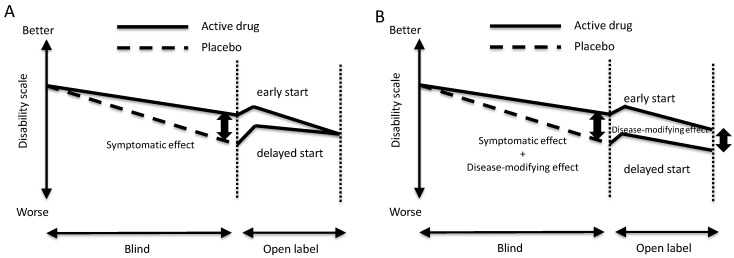
Study design of the delayed-start paradigm to investigate the disease-modifying effects of intervention.

The delayed-start study paradigm was designed to investigate “disease-modifying effects”. As shown in Figure 1, patients are randomly allocated to two groups, namely, early-start and delayed-start groups. Patients in the early-start group are treated with active drug in both blind and open-label periods, and those in the delayed-start group are treated with placebo in the blind period but with active drug in open-label period. At the end of the open-label period, there would be a difference in effects between the early-start and delayed-start groups if the intervention has disease-modifying effects. In contrast, there would be no difference between the two groups if the effects of the intervention are on symptoms, but not disease-modifying. 

Rasagiline, an inhibitor of brain monoamine oxidase, improves the symptoms and signs of Parkinson’s disease. It enhances dopaminergic neuronal transmission because it blocks dopamine degradation by brain monoamine oxidase, and the effects are taken to be symptomatic. Additionally, it may provide neuro-protective effects by inhibition of production of harmful molecules such as methyphenyl pyridinium [16], or promotion of mitochondrial viability [17]. In this context, an open-label rasagiline study following the TEMPO study [18] was performed to investigate disease-modifying effects of rasagiline. It compared motor deteriorations between patients prescribed 2 mg rasagiline for 12 months (early-start group) and those prescribed placebo for 6 months and switched to treatment with 2 mg rasagiline for the following 6 months (delayed-start group). As a result, there was less deterioration of motor functions in the early-start group than in the delayed-start group [10]. To confirm these results, another randomized control trial, the ADAGIO study, was performed. A total of 1,176 untreated Parkinson’s disease patients were enrolled and randomized to early-start and delayed-start groups to investigate the effects of 1 mg and 2 mg rasagiline. The effect of 1 mg rasagiline in the early-start group was superior to that in the delayed-start group; however, there was no significant difference in the effects of 2 mg rasagiline between early-start and delayed-start groups [11]. As described above, the disease-modifying effect of rasagiline is controversial. Though the reason why the result of ADAGIO study was controversial in spite of a large size of the participants has not been fully elucidated, it might be due to the initiation of interventions would be too late to demonstrate disease-modifying effect, even in early-start group.

## 3. Progression and Age of Onset

**Figure 2:**
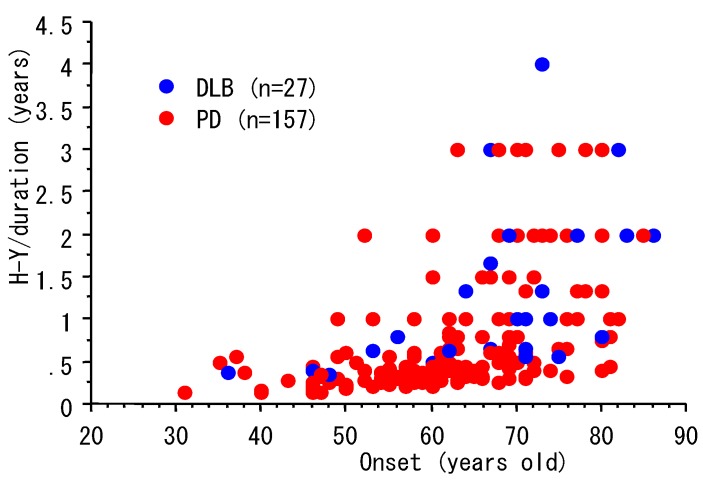
Disease progression and age-of-onset of Parkinson’s disease.

Parkinson’s disease is characterized pathologically by dopaminergic neuronal degeneration and the appearance of Lewy bodies [1,2,3]. These pathological changes are also seen in patients with dementia with Lewy bodies (DLB), in which the motor symptoms progress more rapidly, and dementia and hallucinations occur earlier than in Parkinson’s disease. Compared with Parkinson’s disease, the age-of-onset of DLB is older [19], suggesting that age-of-onset could be associated with the progression of Lewy body pathology. We investigated the relationship between age-of-onset, duration and Hoehn-Yahr stage in 157 Parkinson’s disease patients and 27 DLB patients. Demographic data of the study participants were shown in Table 1. As shown in Figure 2, in patients with an age-of-onset of 50 or younger, it took over 2 years to show a worsening of Hoehn-Yahr stage by 1. In contrast, the disease progressed by 1 or 2 stages within 1 year in a lot of the patients with an age-of-onset of 70 or older. These data suggest that the disease progresses rapidly in patients with later disease onset. Because the prevalence of Parkinson’s disease increases with aging of the population, an early diagnostic test is required for appropriate medical treatment.

**Table 1:** Demographic data of study subjects for relationship disease onset age and disease progression.

	n	Male, n (%)	Age, Y (mean, SD)	duration, Y (mean, SD)
PD	157	72 (46%)	69.8 (8.9)	7.4 (5.9)
DLB	27	15 (56%)	72.5 (9.6)	4.8 (3.5)

PD: Parkinson’s disease, DLB: dementia with Lewy bodies, SD: standard deviation.

## 4. Olfaction and Parkinson’s Disease

In Alzheimer’s disease, olfactory identification is disturbed in the early stage of the disease [20,21]. Olfaction may also be altered in the early stage of Parkinson’s disease [22,23]. The results of pathological analysis have led to the hypothesis that Lewy body pathology develops in the medulla oblongata, pontine tegmentum and olfactory bulb in the early stage, and that it later spreads to the midbrain [24]. In this context olfactory disturbance could precede the symptoms and signs of the motor disturbance in Parkinson’s disease. Ponsen and colleagues showed that hyposmic individuals have a higher risk of developing Parkinson’s disease than the normal population. These authors analyzed the risk of relatives of patients with Parkinson’s disease developing the disease within 2 years, focusing on olfactory function [12]. Olfactory disturbance is one of the earliest non-motor features, seen in 90% of patients of early stage patients, and therefore, can precede motor symptoms [25,26]. Furthermore severe olfactory disturbance could be a prodromal condition of the cognitive decline in Parkinson’s disease [27]. 

All three qualities of olfaction, identification, threshold, and discrimination is disturbed in patients with Parkinson’s disease [25], and they can be examined using “Sniffin’s Stick” test kit (pheylethyl alcohol threshold, odor discrimination and odor identification). Odor identification tests are usually used as a diagnostic test; an odorant is presented at concentrations above the threshold, and then the subject is requested to identify the odor from the written list. The results are dependent on verbal performance and odorants must be well-known to subjects [25].

## 5. Cardiac Denervation and Parkinson’s Disease

Lewy bodies, a pathological hallmark of Parkinson’s disease, are intracytoplasmic inclusions consisting of insoluble aggregated proteins. These include alpha-synuclein, alpha-synuclein-associated proteins (such as symphilin-1), ubiquitin, proteins of the ubiquitin-proteasome system (proteasomes, proteasome activators, and ubiquitinating enzymes), and molecular chaperones [28,29]. Lewy bodies are found in brain neurons such as mesencephalic dopaminergic neurons, noradrenergic neurons in the locus ceruleus, and cholinergic neurons in the nucleus basalis of Mynert; they are also seen in sympathetic ganglia of the peripheral nervous system. Iwanga *et al.* reported cardiac denervation in patients with Parkinson by histological examinations in 1999 [30] and Orimo and colleagues investigated cardiac sympathetic nerve terminals in the epicardium and found that they are lost in patients with Parkinson’s disease [31,32]. Goldstein and his colleagues reported cardiac sympathetic denervation using ^18^F-fluorodopamine scintigram [33]. Meta-iodine-benzyl guanidine (MIBG) is a norepinephrine analogue that is taken up into the sympathetic nerve terminals via the norepinephrine transporter. Cardiac scintigrams using MIBG labeled with ^123^iodine show nerve terminals in the epicardium. Reduction of cardiac MIBG accumulation can be a laboratory marker to differentiate Parkinson’s disease from multiple system atrophy [34,35,36]. When a planar scintigram image of the chest is obtained 40 min (early image) or 4 h (late image) after intravenous ^123^I-MIBG injection and regions-of-interest (ROIs) are set on the heart and upper mediastinum, the relative radio-labeled iodine count ratio of the heart to the mediastinum (early and late H/M ratios) is regarded as the density of cardiac sympathetic nerve terminals. We investigated MIBG cardiac scintigrams of patients presenting Parkinsonism to clarify the diagnostic accuracy. We enrolled consecutive 385 patients who underwent ^123^I-MIBG cardiac scintigram because of muscular rigidity, hand or leg tremor, or slowing movement in two referral hospitals, Utano National Hospital (June 2001 to September 2006) and Kyoto University Hospital (November 2004 to June 2005), a subgroup of the cohort that was published previously [14]. Here, demographic data of subjects and analytic methods are described briefly because detailed data were published [14]. Detailed history was taken and neurological examinations were performed by neurologists and 253 of the 385 patients were diagnosed as definite Parkinson disease according to UK Parkinson’s Disease Society Brain Bank Clinical Diagnostic Criteria (step 2 and step 3). Additionally, we confirmed that no abnormal findings suggesting multiple system atrophy were detected in all of the 253 patients. Among the rest 132 patients who did not fulfilled the UK Brain Bank Criteria of Parkinson disease, 76 patients have typical features for diseases other than Parkinson disease. They were diagnosed as suffering from multiple system atrophy (n = 36), progressive supranuclear palsy (n = 15), essential tremor (n = 12), Alzheimer disease with extrapyramidal signs (n = 5), corticobasal degeneration (n = 3), stroke (n = 3), drug-induced Parkinsonism (n = 1), and motor neuron disease (n = 1). Other 56 of 132 patients were diagnosed as having extrapyramidal sings but were not diagnosed as a specific clinical diagnosis. According to the grades of Hoehn-Yahr in “on-period”, 253 patients with Parkinson disease were separated into 5 groups, grades I (n = 12), II (n = 45), III (n = 62), IV (n = 79), and V (n = 15). The 40 patients were excluded from the subgroup analysis because the grade of Hoehn-Yahr was uncertain. Receiver-operator characteristic (ROC) curves were obtained and the sensitivity and specificity of diagnosis of Parkinson’s disease by MIBG cardiac scintigram were calculated (Table 2 (A,B)). According to the ROC curves, the most discriminating cut-off points of H/M ratios were 1.89 and 1.79 for early H/M and late H/M, respectively, and the sensitivity and specificity were 79.8% and 84.1%, respectively, for early H/M, and 86.6% and 85.6%, respectively, for late H/M. In the subgroup with disease duration of 3 years or less, the sensitivity and specificity were 64.4% and 86.4%, respectively, for early H/M, and 73.3% and 84.1%, respectively, for late H/M. Thus, cardiac MIBG H/M is a good diagnostic marker for Parkinson’s disease and, even in early stage of the disease, a reduction of cardiac MIBG accumulation is a specific finding for Parkinson’s disease. Comparing the results of the MIBG test and olfaction test in terms of their abilities to differentiate Parkinson’s disease from other symptomatic Parkinsonism, the former has higher sensitivity [37].

## 6. Diagnostic Eye Drop Test for Parkinson’s Disease

**Figure 3:**
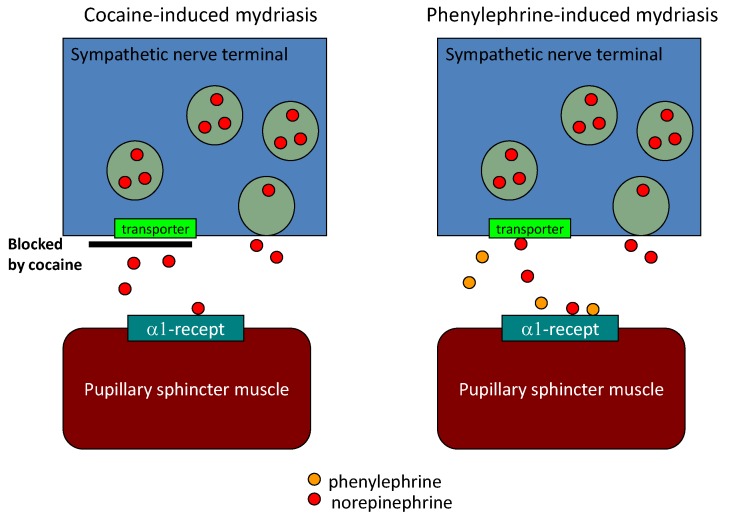
Pupillary mydriasis by cocaine and phenylephrine.

Cardiac sympathetic nerves originate from the cervical sympathetic ganglion that also innervates the pupillary dilator muscle of the eyes. Therefore, as well as sympathetic nerve terminals in the epicardium, those to the pupils could be degenerated in Parkinson’s disease. Cocaine causes mydriasis by blocking norepinephrine uptake. Phenylephrine mimics norepinephrine, and causes mydriasis by attaching to the alpha-1 adrenergic receptor (Figure 3). When the number of sympathetic nerve terminals is reduced, cocaine-induced mydriasis is reduced and phenylephrine-induced mydriasis is enhanced owing to denervation hypersensitivity. In this context, the difference between cocaine-induced mydriasis and phenylephrine-induced mydriasis can be a marker of sympathetic denervation. Although the eye-drop test is non-invasive, low cost, and requires no special apparatus, the results could be influenced by corneal permeability or anterior chamber volume. Previously we reported the diagnostic accuracy of eye drop test using cocaine and phenylephrine in 38 Parkinson’s disease patients, 20 age-matched controls and 10 patients with multiple system atrophy [15]. We compared the mydriasis induced by cocaine and that induced by phenylephrine to cancel these effects. The sensitivity and specificity for diagnosis of Parkinson’s disease based on this parameter are 0.80 (95% confidence interval 0.65–0.94) and 0.79 (0.67–0.93), respectively. The sensitivity and specificity of eye drop test were similar to them of olfactory test [15,38,39] (Table 3). 

Using 18F-fluorodopamine, Tipre *et al.* demonstrated that sympathetic nerve terminals is selectively reduced in the heart and thyroid but not in the liver after adjustment blood perfusion in Parkinson’s disease patient with orthostatic hypotension [40]. Matsui *et al.* also demonstrated reduction of MIBG in the thyroid gland in patients with Parkinson’s disease [41] These data suggest the possibility that the sympathetic nerve terminals that are disturbed originate from paraspinal sympathetic ganglia and travel to the organs; in contrast, those that are preserved originate from nerve ganglia that are located near the organs, and suggest that long nerve terminals may be fragile in the disease (Table 4).

Table 2

**Table:** (**A**) Diagnostic significance of early H/M of diagnosis of PD.

	severity	non-PD/PD	cut-off	Sn	(95% CI)	Sp	(95% CI)	AUC of ROC	(95% CI)
Hoehn and Yahr	I	132/12	<2.12	75.0	(42.8–94.5)	74.2	(65.9–81.4)	0.80	(0.67–0.92)
	II	132/45	<1.88	60.0	(44.3–74.3)	84.1	(76.7–89.9)	0.81	(0.75–0.88)
	III	132/62	<1.86	77.4	(65.0–87.1)	84.9	(77.6–90.5)	0.88	(0.83–0.93)
	IV	132/79	<1.82	87.3	(78.0–93.8)	86.4	(79.3–91.7)	0.90	(0.86–0.94)
	V	132/15	<1.83	86.7	(59.5–98.3)	85.6	(78.4–91.1)	0.93	(0.88–0.97)
Duration of illness (Y)	<3	132/45	<1.81	64.4	(48.8–78.1)	86.4	(79.3–91.7)	0.82	(0.75–0.89)
	3–5	132/61	<1.86	71.1	(59.2–82.9)	84.9	(77.6–90.4)	0.86	(0.81–0.92)
	6–9	132/50	<1.82	76.0	(61.8–86.9)	86.4	(79.3–91.7)	0.89	(0.85–0.94)
	>9	132/62	<1.81	88.7	(78.1–95.3)	86.4	(79.3–91.7)	0.91	(0.87–0.95)
Total		132/253	<1.89	79.8	(74.4–84.6)	84.1	(76.7–89.9)	0.88	(0.84–0.92)

Sn: sensitivity, Sp: specificity.

**Table:** (**B**) Diagnostic significance of late H/M of diagnosis of PD.

	severity	non-PD/PD	cut-off	Sn	(95% CI)	Sp	(95% CI)	AUC of ROC	(95% CI)
Hoehn and Yahr	I	132/12	<2.05	66.7	(34.9–90.1)	72.7	(64.3–80.1)	0.74	(0.60–0.89)
	II	132/45	<2.06	80.0	(65.4–90.4)	72.7	(64.3–80.1)	0.83	(0.76–0.89)
	III	132/62	<1.78	83.9	(72.3–92.0)	86.6	(78.4–91.1)	0.88	(0.83–0.93)
	IV	132/79	<1.67	94.9	(87.5–98.4)	87.1	(80.2–92.3)	0.91	(0.87–0.95)
	V	132/15	<1.56	93.3	(68.1–99.8)	88.6	(82.0–93.5)	0.93	(0.88–0.97)
Duration of illness (Y)	<3	132/45	<1.84	73.3	(58.1–85.4)	84.1	(76.7–89.9)	0.80	(0.73–0.88)
	3–5	132/61	<1.78	80.3	(68.2–89.4)	85.6	(78.4–91.1)	0.87	(0.82–0.92)
	6–9	132/50	<1.85	90.0	(78.2–96.7)	84.1	(76.7–89.9)	0.90	(0.85–0.94)
	>9	132/62	<1.66	96.8	(88.8–99.6)	87.1	(80.2–92.3)	0.92	(0.88–0.96)
Total		132/253	<1.79	86.6	(81.7–90.5)	85.6	(78.4–91.1)	0.92	(0.88–0.92)

Sn: sensitivity, Sp: specificity.

**Table 3:** Non-motor test for diagnosis of Parkinson disease.

Authors	Test modality	Method	LR	Sn (%)	Sp (%)
Doty *et al.* [38]	Olfaction (<59 y.o., male)	UPSIT	6.1	91	85
	(<59 y.o., female)	UPSIT	5.2	79	85
	(60-70 y.o., male)	UPSIT	4.4	81	82
	(60-70 y.o., female)	UPSIT	6.9	80	88
	(≥71 y.o., male)	UPSIT	6.3	76	88
	(≥71 y.o., female)	UPSIT	4.0	74	82
Double *et al.* [39]	Olfaction	B-SIT	3.5	82	77
Sawada *et al.* [15]	Pupillary sympathetic Denervation	Eye drop test	3.9	80	79
Sawada *et al.* [14]	Cardiac sympathetic denervation	MIBG early H/M	5.4	81	85
		MIBG late H/M	8.0	84	90

UPSIT: University of Pennsylvania Smell Inventory Test; B-SIT: Brief Smell Identification Test; LR: likelihood ratio; Sn: sensitivity; Sp: Specificity; H/M: heart to mediastinum ratio.

**Table 4:** Organs and sympathetic nerve terminals.

Organ	Sympathetic Nerve Origin	Sympathetic Nerve Terminals
Heart (epicardium)	Paraspinal sympathetic ganglion	Denervated
Pupil (pupilary sphincter)		
Thyroid gland		
Liver	Celiac ganglion	Preserved
kidney		

## 7. Catecholamines and Dopaminergic Neuronal Death

Although the molecular mechanisms by which brain dopaminergic neurons degenerated have not been elucidated, catecholamines may be a key molecule in the degenerative process. In addition to dopaminergic neurons, and noradrenergic neurons in the locus ceruleus are also degenerated in the brains of patients with Parkinson’s disease. Furthermore, peripheral noradrenergic neurons also degenerate. These findings suggest that neurons that contain catecholamines are fragile in Parkinson’s disease. To explore the role of endogenous dopamine in dopaminergic neuronal death, we investigated the effect of alpha-methyl tyrosine on dopaminergic neuronal death in a rat primary midbrain neuronal culture. Alpha-methy-tyrosine is an inhibitor of tyrosine hydroxylase and depletes intracellular dopamine content. Glutamate is an excitatory neurotransmitter in the striatum that can elicit neuronal death via oxidative stress. A lot of pathological investigations have shown the presence of oxidative stress in Parkinson’s disease [42,43,44]. Using a rat primary midbrain culture, we showed that glutamate-induced dopaminergic neuronal death is blocked by endogenous dopamine depletion [45]. These data suggest that endogenous dopamine could elicit dopaminergic neuronal death. Dopamine quinones, metabolites of dopamine, are supported to interact with α-synuclein and modify ultrastructure of α-synuclein [46]. Aggregation of α-synuclein, a major proteinous component of Lewy bodies, plays a pivotal role in dopaminergic neurodegeneration. Soluble monomeric α-synuclein is not harmful, but partially aggregated forms are harmful to neurons. Dopamine facilitates partial aggregation of α-synuclein [47,48,49,50]. 

## 8. Summary

Dopaminergic neuronal degeneration develops several years before Parkinson’s disease symptoms start, and an early biological marker for diagnosis is required. In Parkinson’s disease, noradrenergic neurons in the peripheral sympathetic nervous system, as well as catecholaminergic neurons including dopaminergic and noradrenergic neurons in the brain, degenerate. Peripheral noradrenergic neuronal degeneration could be an early marker of Parkinson’s disease. Endogenous catecholamines could be a key player in neurodegeneration, because neurons containing dopamine, norepinephrine and serotonin degenerate.
